# Global spread of COVID-19's JN.1 variant: Implications and public health responses

**DOI:** 10.1016/j.nmni.2024.101225

**Published:** 2024-02-05

**Authors:** Prakasini Satapathy, Pawan Kumar, Vini Mehta, Vinay Suresh, Abhinav Khare, Sarvesh Rustagi, Mohammad Naeem Daulati, Mehrab Neyazi, Elyas Najafi, Ahmad Neyazi

**Affiliations:** Center for Global Health Research, Saveetha Medical College and Hospital, Saveetha Institute of Medical and Technical Sciences, Saveetha University, Chennai, India; School of Pharmacy, Graphic Era Hill University, Dehradun, 248001, India; Global Center for Evidence Synthesis, Chandigarh, 160036, India; Department of Dental Research Cell, Dr. D. Y. Patil Dental College and Hospital, Dr. D. Y. Patil Vidyapeeth, Pimpri, Pune, India; King George's Medical University, Lucknow, India; All India Institute of Medical Sciences, Gorakhpur, India; School of Applied and Life Sciences, Uttaranchal University, Dehradun, Uttarakhand, India; Scientific Affairs, Herat Regional Hospital, Herat, Afghanistan; Afghanistan Center for Epidemiological Studies, Herat, Afghanistan; Afghanistan Center for Epidemiological Studies, Herat, Afghanistan; Afghanistan Center for Epidemiological Studies, Herat, Afghanistan

Dear Editor,

As we enter the fourth year of the COVID-19 pandemic, the relentless evolution of the SARS-CoV-2 virus presents new health challenges worldwide, characterized by the emergence of various subvariants of Alpha, Beta, Delta, and Omicron variants of SARS-CoV-2 (1). Each variant has brought its unique set of challenges, and recently, a strain named JN.1, descending from BA.2.86 ‘Pirola’, has come into the spotlight. Initially identified in the United States, JN.1 has now been recognized as an independent Variant of Interest (VOI) by World Health Organisation [2]. Its rapid spread, particularly in countries struggling with other respiratory illnesses and winter seasons, has escalated global concerns [2,3].

What sets JN.1 apart is its additional spike protein mutation. Unlike its close relative BA.2.86, JN.1 possesses a unique mutation in its spike protein, crucial in the virus's ability to infect human cells and the primary target of most COVID-19 vaccines. This mutation in JN.1 may increase its infectivity and its potential to evade immune responses [2]. Given this, JN.1 has swiftly become a dominant strain in many countries, outcompeting others strains like BA.2.86, EG.5, and XBB(4).

JN.1 has one extra mutation in its spike protein (39 spike proteins) compared to its parent strain Pirola (38 spike proteins) and differs significantly from the earlier Omicron XBB.1.5 variant. This additional mutation (L455S) in the receptor binding domain has rapidly made it the dominant strain [1]. In recent weeks, JN.1 has outcompeted several other variants, including its parent BA.2.86, as well as EG.5 (Eris), and XBB.1.16 (Arcturus) [4,5]. This characteristic of JN.1 necessitates further research to explore how JN.1's mutations may impact the course of the pandemic and the effectiveness of current vaccines and treatments.

Fig. 1 explains the genetic basis of how viruses evolve over time through mutations in their genome, which can significantly impact their characteristics and how they interact with host organisms, including humans. Understanding these mechanisms is crucial for developing effective strategies to combat viral diseases.

**Fig. 1:**
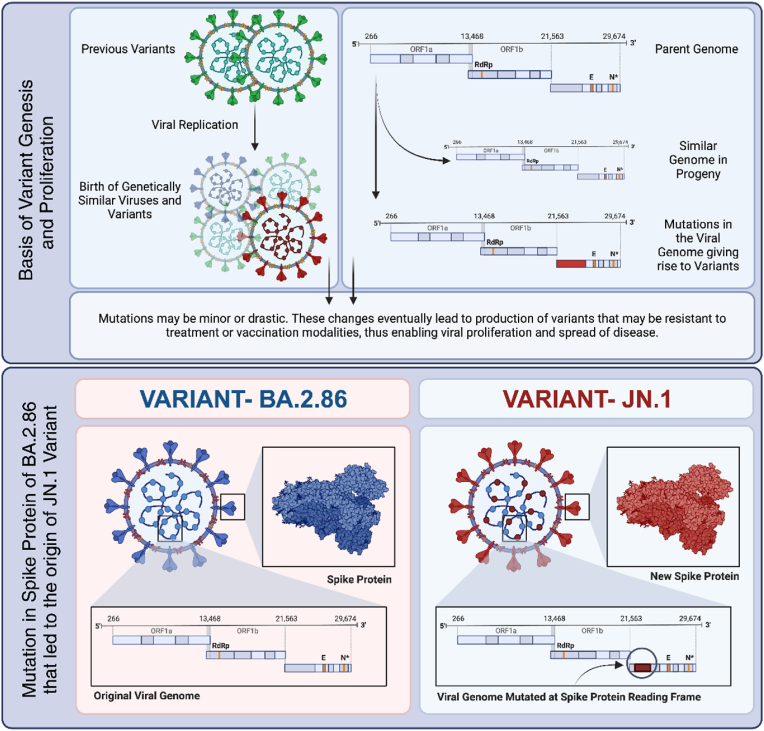
Mechanisms of viral mutation and the emergence of new variants [created with BioRender - https://www.biorender.com].

Globally, JN.1's impact is visible. Documented in over 41 countries, it has become the fastest-growing variant in regions like the United States, France, Singapore, Canada, the United Kingdom, and Sweden. Notably, Singapore has seen a surge in COVID-19 cases driven by JN.1, and its emergence in India has caused significant concern [2].

The JN.1 variant presents symptoms that are common to many respiratory illnesses, making it difficult to distinguish from other conditions like the flu. Symptoms include persistent coughing, sore throat, runny nose, body ache, fatigue, and in some cases, gastrointestinal issues. A critical symptom to watch for is breathlessness, which can be a sign of more severe infection, particularly in older adults and those with weakened immune systems. While mild symptoms can often be managed with symptomatic care, the emergence of more severe symptoms, particularly difficulty in breathing, warrants immediate medical attention [4].

There is a pressing need for tracking new SARS-CoV-2 variants. Public health measures are crucial in controlling the spread of JN.1 strain with the help of widespread vaccination campaigns, mask-wearing, hand hygiene, and practicing social distancing [3]. The early detection and prompt isolation of cases are key to managing the spread of JN.1. This vigilance is vital in crowded settings where the risk of transmission is higher [2]. Furthermore, the use of laboratory investigations such as RT-PCR and antigen testing is essential in accurately tracking and responding to the evolving situation. As the world continues to face the challenges posed by COVID-19, these public health measures remain our best defines against the spread of variants like JN.1 subvariant [4].

The emergence of the JN.1 sub-variant underscores the ever-changing SARS-CoV-2 pandemic. Effective control requires ongoing vigilance, improved surveillance, and public health strategies. Addressing these variant calls for increased research and global collaboration, which are crucial in handling the pandemic's unpredictability.

## Funding

None.

## CRediT authorship contribution statement

**Prakasini Satapathy:** Conceptualization, Writing – original draft, Writing – review & editing. **Pawan Kumar:** Conceptualization, Writing – original draft, Writing – review & editing. **Vini Mehta:** Conceptualization, Writing – original draft, Writing – review & editing. **Vinay Suresh:** Conceptualization, Writing – original draft, Writing – review & editing. **Abhinav Khare:** Conceptualization, Writing – original draft, Writing – review & editing. **Sarvesh Rustagi:** Conceptualization, Writing – original draft, Writing – review & editing. **Mohammad Naeem Dawlati:** Conceptualization, Writing – original draft, Writing – review & editing. **Mehrab Neyazi:** Conceptualization, Writing – original draft, Writing – review & editing. **Elyas Najafi:** Conceptualization, Writing – original draft, Writing – review & editing. **Ahmad Neyazi:** Conceptualization, Writing – original draft, Writing – review & editing.

## Declaration of competing interest

The authors delcare no conflict of interest.
